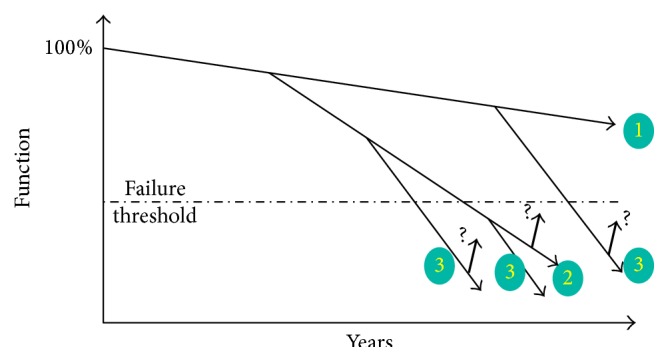# Successful Cognitive Aging: Between Functional Decline and Failure of Compensatory Mechanisms

**DOI:** 10.1155/2015/367407

**Published:** 2015-11-26

**Authors:** Marc Verny, Emmanuel Moyse, Slavica Krantic

**Affiliations:** ^1^APHP, DHU FAST, Groupe Hospitalier Pitié Salpêtrière-Charles Foix, Centre de Gériatrie, Pierre & Marie University, Sorbonne-Paris-Cité Universities, UMR 8256 CNRS B2A, 47-83 boulevard de l'Hôpital, 75013 Paris, France; ^2^INRA Centre, Physiology and Behavior Unit, University François Rabelais of Tours, 37380 Nouzilly, France; ^3^Centre de Recherche des Cordeliers, Paris-Descartes University, Sorbonne-Paris-Cité, Pierre and Marie Curie University, UMR-S-1138 INSERM, 15 rue de l'École de Médecine, 75006 Paris, France

This special issue is focussed on the nature of “successful brain aging”, as opposed to neuropathological cognitive defects, and the underlying compensatory mechanisms resulting in compensation of age-related dysfunctions in a homeostatic manner.

The biological definition of ageing posits that it consists of “a progressive accumulation of deleterious changes in cells and tissues that increase the risk of disease and death” [1]. For any attempt to assay the impact of aging* per se* on cognition in humans, the major problem lies in the selection criteria for subjects to be included in the study. “Disease-free” or “healthy” old subjects are recruited through public call to volunteers, after their appropriateness has been checked by assessment of the participants' general mental health via a battery of cognitive tests and cerebral MRI. However, it has to be stressed that, clinically, ageing* per se* is not a pathology; in other words, ageing is obligatorily normal. These considerations associated with the mean longevity in developed human societies led to introducing the following categorization among elderly people: young-old (6th-7th decades of age), middle-old (7th-8th decades), and oldest-old (above 85 years). The operational “generic” definition of “old subject” in geriatric medicine is nowadays “dependant or at risk of dependency”; it usually applies above 75 years.

To study the ageing on selected cohorts, two principal methods are used: transversal or longitudinal studies. The “transversal approach” implies comparison between aged and young subjects, as in laboratory animal studies of aging mechanisms at the cell and tissue level [2]. Old animals have the same genome and were bred in the same conditions as the young individuals and though constitute the control references with regard to the ageing process, which is obviously not the case for humans. Young subjects cannot be considered as controls for aging-related features of 50-year-older subjects. The life conditions of old subjects at youth differed from those of subjects recruited as the young controls by numerous aspects impacting health and cognitive performance (diet, war-related stress, cognitive training, and activity). According to the French hospital statistics indeed, the subjects admitted in geriatric departments in 1990 had a 15-year lower mean age and were less healthy than those admitted in 2015. This bias is known as the cohort effect. Therefore, the more accurate analysis of ageing relies on a “longitudinal approach” in a cohort of subjects, using regularly spaced examinations across the ageing duration, that is, two-three decades. This implies a progressive attrition of the studied cohort, because of personal moving, decease, and disease onset that causes the case exclusion. This is the bias of attrition known to select preferentially the less dependent persons. In summary, transversal methods tend to exaggerate the effect of ageing and longitudinal methods tend to minimize it.

To define the impact of ageing on cognitive functions, that is, the average evolution of cognitive performances “as a function of chronological age in disease-free humans,” which is frequently termed “across normal ageing,” we need to define what is “normality” among old humans and especially with regard to cognition [3]. Ageing is commonly recognized to include a progressive decline of physiological efficiency in all organs, which can be schematized as a negative slope in the graphical plot of function index against age increase [4]. Medical observations show that, for all organs, clinical manifestations of deficiency appear when this slope crosses 30–50% of maximal (100%) values of optimal subject's functionality; this is termed “failure threshold.” This may be also applicable to the nervous system. In Parkinson's disease thus, the first clinical symptoms seem to appear when 30–70% of the mesencephalic nigrostriatal dopamine neurons are lost [5]. These data suggest that the organs and functions encompass a functional reserve. However, the age-related negative slope of physiological functionality displays interindividual variations that are more and more expressed as chronological age advances. Some subjects in the sixties of age suffer much heavier physiological deficiencies than most of older ones. Therefore, a strictly chronological categorization of elderly humans is excessively reductionist. In particular, some subjects remain free of any chronic disease and display little or none physical or intellectual limitations up to advanced ages, which has been defined as “successful ageing” [6]. A more numerous part of elderly has some physical or intellectual limitations without real handicap and can be classified as “usual ageing.”

To provide better comprehension of the complexity of an old individual, a French author proposed analysis of geriatric medical situations following a rule including 3 axes (see [7], Figure 1). Axis 1 represents the physiological ageing as a decline of the capacity to adaptation or reduction of a “static reserve” but this slope never attains the level of incapacity. Axis 2 represents the chronic diseases like Alzheimer's. This axis has a part upstream of the dotted line representing the deficiency (i.e., dementia in this case) which may correspond to the prodromal period of the Alzheimer's disease (AD). Axis 3 represents an affection (usually acute) which is totally ageing-independent but exacerbates the deleterious impact of factor 2. Regarding clinical studies of ageing impact on cognitive functions, since AD is usually diagnosed at late stages only, any group of “disease-free” aged subjects will include a number of prodromal AD patients whose memory performances are already weaker than healthy subjects. This fact strengthens the advantage of the longitudinal approach as compared to the transversal one.

Bouchon's axis 1 (Figure 1) can be influenced by genetic or environmental factors. Genetic factors include accumulation of genes that provide predisposition to longevity and though could be protective against cognitive decline across ageing. As a corollary, axis 1 may correspond to* the cognitive reserve concept*, which is born of observations demonstrating that some patients appear more resistant to the brain pathology, such as AD. Indeed, the dementia incidence is significantly lower in subjects with higher educational level and diplomas than in the general population [8]. Among the environmental factors affecting Bouchon's axis 1, longitudinal cohort study [8] indicated that the practice of leisure activities demanding planification, such as gardening, traveling, odd jobs, and knitting, decreases the risk of dementia [9]. This notion has been strengthened in the “Nun study” by correlating educational level with the results of regular neuropsychological tests [10]. It turned out that, in the global nun population, the cognitive impairment was significantly correlated with the severity of AD neuropathology, as quantified in postmortem brains by Braak's staging. By contrast, higher literary quality of autobiographies has been correlated with decreased risk of dementia [11]. However, a small subgroup displayed Braak's stages V-VI with intact memory and mild cognitive impairment, that is, AD histopathology in advance over cognitive decline, suggesting that these subjects (displaying the highest cognitive reserve) were spared [10]. An early and sustained practice of the most complex cognitive functions would thus allow building up reserves against ageing-related cognitive declines. In this light, cognitive reserve represents the capacity of individuals to resist cognitive alterations, including neuropathological ones. This concept has been further supported by a recent analysis of cognitive impairments among a population of AD patients [12]. This analysis indicated that when the cognitive reserve is weak, decline manifestations appear more progressively and slower than in subjects with high cognitive reserve [13], probably because in subjects with low cognitive reserve decline appears when lesion level is already very high. Obvious question is how to define and measure cognitive reserve for clinical purposes, how to achieve that most of people acquire the highest cognitive reserves, and how to prolong utilization of these reserves.

Bouchon's “factor 2” (Figure 1) reflects the breakpoint in the cognitive decline curve corresponding to clinically diagnosed AD, corresponding to around 70 years of age. This is precisely the age category that is currently increasing the most, according to demographic statistics [14]. The 70-year-old population encompasses a large proportion of subjects displaying* mild cognitive impairment* (MCI), that is, who complain about memory impairments but do not meet clinical criteria of AD in the absence of significant influence on daily living activities. Previous longitudinal studies have revealed that above 50% of MCI subjects convert into AD within 5 years, suggesting that MCI represents a predementia (or prodromal) stage of the disease rather than physiological ageing [15]. The chronological relationship between prodromal stage and demented stage of AD has been evaluated in the PAQUID study [8]. Retrospective analysis performed in this study showed that subjects who had been diagnosed with dementia of AD displayed significant cognitive alterations (as compared to those without diagnosis of AD) around 13 years before the onset of clinically defined AD [16]. The remaining subjects of the cohort yielded a mean cognitive decline curve which was significantly different from the mean cognitive decline curve of the latent AD subpopulation. This led to updating AD concept and proposing a new set of diagnostic criteria for the research [17]. On these bases, any aged person who complains about memory deficits should be examined for biological diagnostic of MCI using CSF biomarkers or PET scan. Among the MCI patients that are identified as prodromal state of AD with the help of these new markers, those with high risk to develop AD are distinguished from those at lower risk by the highest cognitive reserve.

In keeping with Bouchon's rule, additional pathologies impinging on brain functions could precipitate the evolution from prodromal stage of AD to dementia. The majority of clinical dementia cases among elderly people are indeed accounted for by mixed brain pathologies rather than pure neuropathologies [18]. Bouchon's “factor 3” (Figure 1) in cognitive ageing corresponds to interference of acute pathologies (infectious, metabolic, traumatic, etc.) that render a person at the prodromal stage of AD more susceptible to experiment a delirium [19].

The contributions in this special issue deal with all of the above discussed aspects of aging. The contribution by M. Suwa et al. (Takatsuki Hospital, Osaka, Japon) discusses the predictive value of cerebral white matter intensity for cognitive impairment in neurologically “healthy” subjects displaying at least one atherosclerosis risk factor (age range 55–84 years) and how dietary intervention by sufficient polyunsaturated fatty acids intake may positively impact on the cognitive impairment. The work by Y. Os et al. from Maastricht University (Netherlands) focuses on cognitive intervention strategies and shows that these strategies change the brain activation process even in cognitively impaired persons; whether such cognitive interventions may be effective to delay conversion to dementia remains an open question. In a transversal study of neurologically “normal” young (18–35 years), mid-aged (36–55 years), and old (56–75 years) subjects, L. R. Demenescu et al. from the University Hospital of Aachen (Aachen, Germany) report how emotion recognition deficits emerge with increasing age. E. Bauer et al. from the University of Giessen (Giessen, Germany) and Evangelic Hospital (Bielefeld, Germany) assessed how the level of cognitive performance may impact on task load within left rostral prefrontal cortex; their data indicate that both parameters (age and performance level) affect the load-dependent activation within rostral prefrontal cortex. M. Silagi et al. (University of São Paulo “São Paulo, Brazil”) present the evidence on the central mechanisms which compensate for the auditory deficits in sentence comprehension during the course of the normal aging. A.-M. Kirova et al. (Skidmore College, USA) highlight the mechanisms relevant to working memory and executive function decline across normal aging, MCI, and Alzheimer's disease. As the general light-motive of all these studies, it appears that cognitive ageing consists likely much more in a decrease of cognitive reserve than in functional losses.

## Figures and Tables

**Figure 1:**